# Direct Writing of Flexible Electronics through Room Temperature Liquid Metal Ink

**DOI:** 10.1371/journal.pone.0045485

**Published:** 2012-09-19

**Authors:** Yunxia Gao, Haiyan Li, Jing Liu

**Affiliations:** 1 Key Laboratory of Cryogenics, Technical Institute of Physics and Chemistry, Chinese Academy of Sciences, Beijing, China; 2 Department of Biomedical Engineering, School of Medicine, Tsinghua University, Beijing, China; Texas A&M University, United States of America

## Abstract

**Background:**

Conventional approaches of making a flexible circuit are generally complex, environment unfriendly, time and energy consuming, and thus expensive. Here, we describe for the first time the method of using high-performance GaIn_10_-based electrical ink, a significantly neglected room temperature liquid metal, as both electrical conductors and interconnects, for directly writing flexible electronics via a rather easy going and cost effective way.

**Methods:**

The new generation electric ink was made and its wettability with various materials was modified to be easily written on a group of either soft or rigid substrates such as epoxy resin board, glass, plastic, silica gel, paper, cotton, textiles, cloth and fiber etc. Conceptual experiments were performed to demonstrate and evaluate the capability of directly writing the electrical circuits via the invented metal ink. Mechanisms involved were interpreted through a series of fundamental measurements.

**Results:**

The electrical resistivity of the fluid like GaIn_10_-based material was measured as 34.5 µΩ·cm at 297 K by four point probe method and increased with addition of the oxygen quantity, which indicates it as an excellent metal ink. The conductive line can be written with features that are approximately 10 µm thick. Several functional devices such as a light emitting diode (LED) array showing designed lighting patterns and electrical fan were made to work by directly writing the liquid metal on the specific flexible substrates. And satisfactory performances were obtained.

**Conclusions:**

The present method opens the way to directly and quickly writing flexible electronics which can be as simple as signing a name or drawing a picture on the paper. The unique merit of the GaIn_10_-based liquid metal ink lies in its low melting temperature, well controlled wettability, high electrical conductivity and good biocompability. The new electronics writing strategy and basic principle has generalized purpose and can be extended to more industrial areas, even daily life.

## Introduction

The application of flexible electronics is growing rapidly, as promoted by a group of newly emerging areas. In recent years, many investigators have been attracted and tremendous efforts were made along this direction. Breakthroughs thus made will benefit the rapid development of modern consumer electronics including flexible displays [1]–[3], conformal antenna arrays, thin film transistors [4]–[7], membrane keyboards, electronic solar cell arrays [8], [9], radio-frequency identification (RFID) tags [10], flexible batteries [11], [12], and electronic circuits fabricated in e.g. clothing or biomedical devices etc. [13]–[16]. The flexible electronics represent a number of highly desired features for customers, such as comfortable wearing, cost reduction, improved robustness, bendable properties and lower power consumption etc. It is forecasted that the market for flexible electronics will increase as large as that of the current Si electronics. However, the conventional electrical circuit fabrication technologies are subject to limitations in that they request multistep, involve high processing temperatures and toxic waste and are therefore more expensive. What is more important is that the high processing temperature restricts the application of the flexible substrates, such as plastic and other polymer materials. Hence, the directly printing techniques such as nanoimprinting [17], screen printing [18] and ink jet imprinting [19]–[21] have emerged as attractive direct patterning techniques and promoted a big development in flexible electronics fabrication fields.

In addition, conventional printed circuits and electrical interconnects are generally composed of electrochemically etched copper foils and eutectic Sn/Pb solders [22]. Although with many advantages, the high processing temperature and lead toxicity, as well as the low fabrication speed severely hurdle their broader applications in future flexible electronics [23], especially in those areas like wearable devices or textile industries. Lately, there have been amounts of studies into flexible electronic devices which are targeted for reducing material cost, decreasing processing temperature and improving fabrication speed [24]–[26]. Cao *et al.* formed a carbon nanotube thin-film integrated circuits composed of up to nearly 100 transistors on flexible sheets of plastic [27]. In addition, the carbon nanotube ink is also printed on the flexible substrate to fabricate simple high-frequency electronic devices such as resistances, capacitances or inductances [28]. Meanwhile, some other researchers turned to paper substrates which can be used as a low-cost, enabling platform for flexible and disposable devices [29], [30]. In fact, paper substrates offer many advantages for printed electronic devices. Not only is paper widely available and inexpensive, it is lightweight, biodegradable, and can be rolled or folded into 3D configurations. As a result, functional electronic components have recently been produced on paper substrates. Russo *et al.* demonstrate a facile pen filled with silver ink which can be written on paper [31], and a 3D antenna has therefore been fabricated by drawing periodic conductive silver tracks on a sticky paper. However, the cost of the silver is pretty high and the procedure for preparing the compound silver ink is still relatively complex and time consuming.

Different from all the previous electronics making strategies, here we proposed for the first time to realize the direct writing of flexible electronics based on room temperature liquid metal. The electrical ink comes from the gallium and its alloys, whose direct applications were significantly neglected until now. Compared with the conventional silver chemicals, such materials own many unique favorable virtues such as having low melting point, being directly printable, conductive, biocompatible, low cost and so on. The present electrical ink can be easily and quickly printed as electrical conductors and interconnects, even functional devices for flexible electronics. This makes the realization of a circuit as simple as that writing a word or drawing a picture on the paper.

A critical difficulty for one to overcome in the present method is to improve the wettability between the room temperature liquid metal and the specific materials. Hence in this paper, GaIn_10_ alloy with a lower melting point than gallium was adopted as raw material and controlled oxidation reaction was carefully employed aiming to modify its wettability. The comprehensive experiments demonstrate for the first time that the GaIn_10_-based liquid metal ink shows an excellent wettability with a wide range of materials because of the existence of gallium oxides. For further characterizing the adhesion of the GaIn_10_-based liquid metal ink, a series of experiments monitored by high speed camera have been carried out. Scanning electron microscope (SEM) was applied to observe the surface morphology and thickness of conductive line written on paper. In addition, the electrical properties of GaIn_10_-based liquid metal ink were studied by four point probe method. Meanwhile, experiments were also performed to directly write conductive text, interconnects for light emitting diode (LED) arrays and electrical fan on flexible substrates. This new method opens many important potentials in quickly designing flexible electronics. Its application is not only in the industries, but can also be widely adopted in educational area, such as that university, middle or even elementary school teachers can teach students the first hand knowledge of electric circuits using the present liquid metal ink.

## Materials and Methods

### Preparation of GaIn_10_-Based Liquid Metal Ink

Gallium and indium metals with purity of 99.99 percent used as raw materials were weighted with a weight ratio of 90∶10 according to the chemical composition of GaIn_10_ alloy. The detailed synthesis procedures were outlined respectively as follows:

Firstly, the weighted gallium and indium were added into beaker and heated at 373K to prepare GaIn_10_ alloy. The mixture were stirred using a magnetic stirrer when they were all melted. Then 10 ml 30% NaOH solution was added slowly to clean the metals. The mixture was stirred at room temperature for short periods of time. The contents of the beaker had both an aqueous phase and a metallic phase. Then, the GaIn_10_ alloy was separated from the mixture and stirred constantly in air at room temperature to be oxidized. The oxide layer formed easily on the surface of alloys was broken by the vigorous stirring so that more and more gallium metal can be oxidized. With the increase of gallium oxides formed, the viscosity of alloy will be increased.

It was worth to mention that the experimental parameters such as amounts of GaIn_10_ alloy, stir time and stir speed should be tightly controlled. In these experiments, the weight of GaIn_10_ alloy was chosen to be 40g and stir speed was chosen as 200 r/min, hence it is easy to control accurately the percentage of oxygen which was adjusted accurately by controlling the stirring time. After being vigorously stirred for 5min, 10min, 20min and 30min respectively, one can get the GaIn_10_-based liquid metal inks with different viscosity, which was composed of GaIn_10_ alloy and different percentage of gallium oxide dispersed uniformly in alloy. The quantity of oxygen can be calculated by the weight increase of GaIn_10_-based liquid metal with and without oxidization. All the experiments were made at a relative humidity level of 71.8%.

### Characterization of GaIn_10_-Based Liquid Metal Ink

The surface morphology and thickness of conductive line written on papers were observed with field-emission scanning electron microscope (FESEM, S4300, Hitachi). Flowing process of metal ink droplet on paper were recorded by a high-speed camera (NR4-S3, USA-IDT) with a detection velocity of 500fps. Electrical resistivity measurements were performed using four point probe method in air at 5 different temperatures which were measured with an accuracy ±0.5^o^C by T-type thermocouples and the liquid metal inks were encapsulated into the 14# silicone tube with inner diameter of 1.6 mm to avoid the affection of water. All the data were recorded using Agilent 34972A, USA. Prior to each measurement, the sample was stabilized at constant temperature for at least 2h to ensure thermal equilibrium.

## Results

### Component Analysis on the Metal Ink

**Figure 1:**
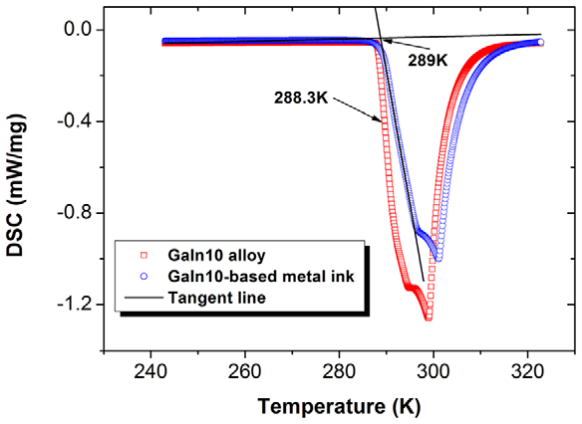
The differential scanning calorimeter curve of GaIn_10_ alloy (weight percentage, Ga90%, In10%) and GaIn_10_-based liquid metal ink which consists of 0.026 wt% oxygen.

GaIn_10_-based liquid metal ink has been prepared by controlling an oxidization reaction of GaIn_10_ alloy (weight percent, Ga90%, In10%). Figure 1 shows the differential scanning calorimeter curve of GaIn_10_ alloy and GaIn_10_-based liquid metal ink which consists of 0.026 wt% oxygen. It can be observed that they all have a lower melting point of 288.3K for GaIn_10_ alloy and 289K for metal ink, respectively. Comparing with GaIn_10_ alloy, there is a slight increase of melting point for metal ink which may be associated with the measurement error. In addition, Gallium alloys have been tested to have a higher boiling point which makes it in liquid state even higher than 2000^o^C [32].

So far, there is still no experimental condition to investigate the crystal structure of GaIn_10_ alloy and GaIn_10_-based liquid metal ink in our lab because of their liquid state at room temperature. However, Szymanski *et al.* have studied that the Ga-In alloy crystallizes in Orthorhombic and the space group is Cmcm [33]. And it has been proved that the existence of a very small amount of gallium oxides does not change the crystal structure of the liquid metal [34]. Hence the XRD patterns of GaIn_10_ alloy and GaIn_10_-based metal ink may be similar with Ga_3.84_In_0.16_ (JCPDS 33-0556) and Ga_0.9_In_0.1_ (at.%) (JCPDS 34-1437).

**Figure 2:**
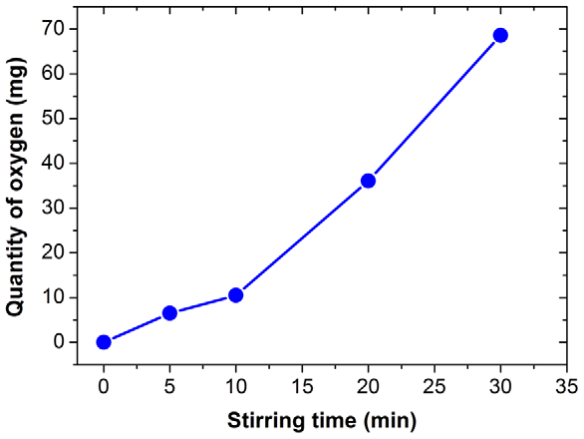
The content of oxygen as a function of stirring time. The quantity of oxygen can be calculated by weighting 40g GaIn_10_ liquid metal with and without oxidation.

After being vigorously stirred for different time period (5 min – 30 min), the GaIn_10_-based liquid metal ink consisting of various quantity of oxygen have been prepared. Figure 2 shows the quantity of oxygen as a function of stirring time. The content of oxygen contained in the metal ink is larger as stirring time increases. Further increasing the stirring time, the GaIn_10_-based liquid metal ink will become thicker and more viscous so that it may not be beneficial to be written on substrate. Hence selecting an appropriate content of gallium oxides is a key problem.

### Disclosing the Role of Oxides in Modifying Wettability of the Metal Ink

**Figure 3:**
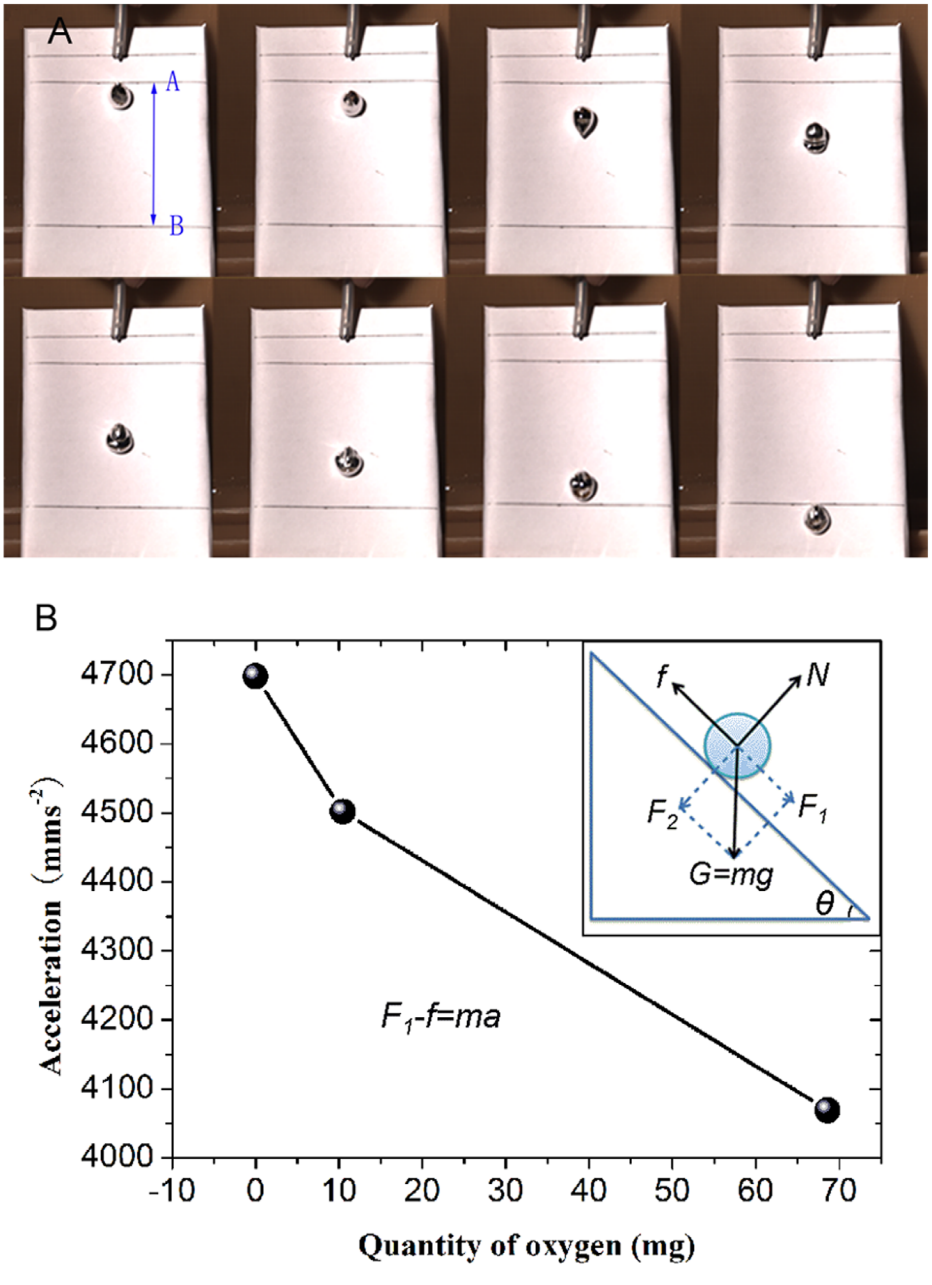
Characterizing the viscosity of GaIn10-based liquid metal ink flowing on the paper with a slope angle of 45^o^ through high-speed camera. (**A**) Optical image of liquid metal ink flowing on the paper. (**B**) Acceleration variation of the droplet as a function of quantity of oxygen in 40g GaIn_10_-based liquid metal ink, and the inset shows the sketch map of force analysis of the droplet in the flowing process.

GaIn_10_-based liquid metal ink has a much higher viscosity. Here, for the characterization of the relative viscosity of GaIn_10_-based liquid metal ink with different quantity of gallium oxides, a high-speed camera is particularly employed to record the flowing process of each liquid metal ink droplet on the paper with a slope angle of 45^o^. For a comparison, the volume of each ink droplet with different content of oxides should be the same. Hence in this paper the droplets are generated by an injector pump, the injection rate is 10ml/h and the volume of each droplet is about 270 µl. Figure 3A shows the detailed flowing process of a liquid metal ink droplet and for a comparative evaluation, each droplet should be running a same distance from A to B. Figure 3B presents the acceleration variation of the droplet as a function of quantity of oxygen and the inset shows the sketch map of force analysis of the droplet in the flowing process. It can be seen that there are mainly three forces which are acting on the droplet, such as gravity (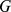), supporting force (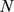) and friction (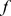). The speed of each droplet is increasing in the flowing process, according to the sketch map of force analysis of the droplet, Newton second law can be used to describe the movement of the droplet. The basic equation can be approximately written as follows:
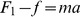
(1)


where, 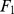 is the gravity component which is parallel to the flowing plane; 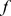 is the friction which increases as liquid metal ink viscosity increases; 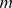 and *a* are the quality and acceleration of droplet, respectively.

So, the acceleration of droplet can be described as:
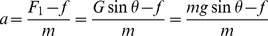
(2)


where, 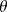 is the slope angle of the plane. In addition, for liquid metal ink droplets with different content of oxides, 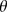 and 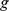 are all constants, the variation of *m* derived from the different percentage of oxides is so small that it can be ignored because of a little amount of oxides dispersed into the GaIn10-based liquid metal ink. Hence, there are only two variables: *a* and *f*. According to eq 2, it can be seen that *a* decreases as *f* increases. As is well known, with increasing of the relative viscosity of GaIn_10_-based liquid metal ink having different percentage of gallium oxides, the friction of liquid metal ink droplet increases. Hence we get the conclusion that the acceleration decreases as the viscosity of droplet increases, which is well proved by the Figure 3B. Here the acceleration is generated by fitting the curve of second degree. It again indicates that a little amount of gallium oxides uniformly dispersed into GaIn_10_-based liquid metal ink will significantly improve the viscosity of GaIn_10_-based liquid metal ink with other materials. Such phenomenon is in good agreement with the conclusion given by Taylor *et al.* in 1998, who pointed out that gallium oxide was readily formed in gallium alloys and can be wetted on different materials [35].

### Electrical Resistivity of Metal Ink with Different Percentage of Gallium Oxides

**Figure 4:**
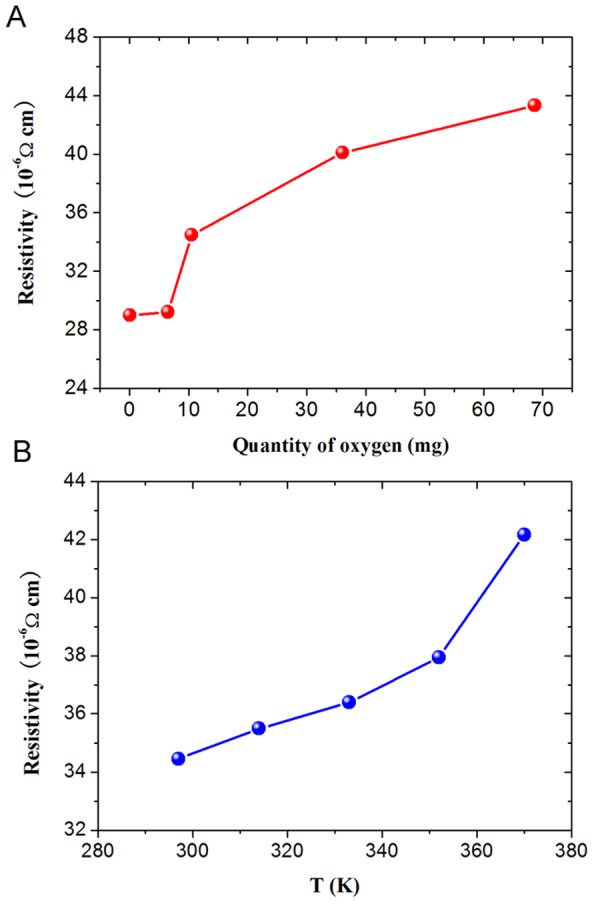
Electrical resistivity of GaIn_10_-based liquid metal ink. (**A**) Electrical resistivity of GaIn_10_-based liquid metal ink as a function of oxygen content contained in 40g GaIn_10_-based liquid metal ink at room temperature. (**B**) Electrical resistivity of GaIn_10_-based liquid metal ink as a function of measuring temperature.

The increase of the oxygen content as contained in the metal ink effectively decreases the surface tension and improves the viscosity of metal ink. However, the oxidized alloys may have a higher electrical resistance [35]. Electrical resistivity as another important factor for fabricating effective electronics was studied by four point probe method. Figure 4 shows the electrical resistivity of metal ink as a function of the oxygen quantity (Figure 4A) and temperature (Figure 4B). It can be observed that the existence of gallium oxides indeed affect the electrical resistivity as shown in Figure 4A. The electrical resistivity of metal ink increases from 29.0 µΩ·cm to 43.3 µΩ·cm at room temperature by increasing the oxygen quantity from 0 mg to 68.6 mg which is contained in 40g GaIn_10_-based liquid metal ink.

As is well known, with the stirred time increasing, the viscosity of liquid metal ink will increase. Here the liquid metal ink with 68.6mg oxides have the biggest viscosity corresponding to the highest electrical resistivity of 43.3 µΩ·cm at room temperature, which cannot be stirred sequentially and keeps at a semi-liquid state with little fluidity. Clearly, the GaIn_10_-based liquid metal serving as the conductive ink written on paper should have a better wettability and lower electrical resistivity. In consideration of the above two factors, the oxidization time in this work has been chosen as 10 min which is corresponding to the fact that metal ink consists of more than 99% liquid metal and only a little mount of gallium oxides equally dispersed in the metal ink. Such metal ink not only can be directly and easily written on papers without any leakage, skipping or flowing, but also presents a very high electrical resistivity of 34.5 µΩ·cm at room temperature as shown in Figure 4A and Figure 4B.

### Wettability Evaluation between Metal Ink and Substrate

**Figure 5:**
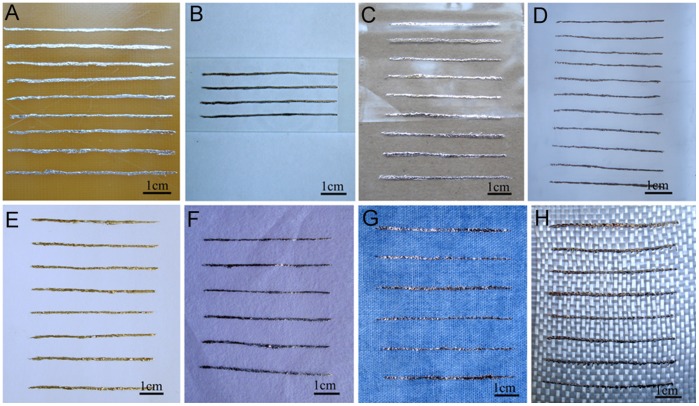
Demonstrated wettability of GaIn10-based liquid metal ink written on different substrate materials. (**A**) Epoxy resin board; (**B**) Glass; (**C**) Plastic; (**D**) Silica gel plate; (**E**) Typing paper; (**F**) Cotton paper; (**G**) Cotton cloth; (**H**) Glass fiber cloth.

GaIn_10_-based liquid metal with only 0.026% oxygen serving as a special metal ink for fabricating effective circuit board has rather important practical significance and broad application prospect. Its wettability with a group of selected substrate materials with either soft or rigid properties which are expected to be used in future electronics, has been studied as shown in Figure 5. It can be found that, given an appropriate modification, the GaIn_10_-based liquid metal ink with only 0.026% oxygen would produce excellent wettability with almost any desired materials including epoxy resin board (Figure 5A), glass (Figure 5B), plastic (Figure 5C), silica gel plate (Figure 5D), paper (Figure 5E), cotton (Figure 5F), cloth (Figure 5G), glass fiber (Figure 5H) and more materials which have the different surface roughness. For example, the roughness of glass (Figure 5B) and the plastic cloth (Figure 5C) are only about a few nanometers, the epoxy resin board (Figure 5A) and silica gel plate (Figure 5D) may reach tens to several hundred nanometers. While the roughness of typing paper and cotton paper is in micron dimension, and the surface of textiles such as cotton cloth and glass fiber cloth are in millimeter range. The better wettability of GaIn_10_-based liquid metal ink with many either soft or rigid materials owning different surface roughness can be well explained by the existence of gallium oxide in the ink [36]. Clearly, the perfect wettability of GaIn_10_-based liquid metal ink makes it possible to use the room temperature liquid metal to directly and easily draw electrodes and electrical conductors with any desired circuit lines and patterns on a desired substrate. In addition, through combination with other materials with varied properties such as super conductive, semi-conductive, optical, magnetic or just isolating, 3D functional electronics can also be realized. More importantly, the 3D functional electronics can be printed on various substrate materials to satisfy the more actual demands in some special applications. This proves the generalized concept of direct writing of flexible electronics, which is expected to be significant in future scientific and industrial fields.

### Direct Writing of Liquid Metal Ink on Paper

**Figure 6:**
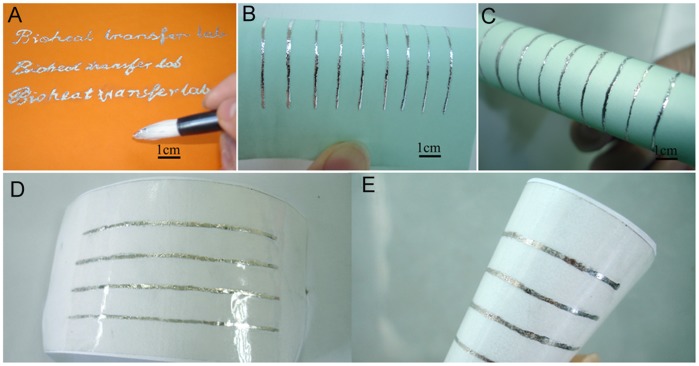
Optical images of GaIn_10_-based liquid metal ink directly written on papers using brush pen. (**A**) Optical image of conductive text written on typing paper by using GaIn_10_-based liquid metal ink. (**B**) and (**C**) Optical images of conductive lines in bent states written on typing paper by using GaIn10-based liquid metal ink. (**D**) and (**E**) Optical images of conductive lines covered with a polyvinyl chloride film.

As demonstrated by our present experiments, the GaIn_10_-based liquid metal ink prepared with oxidation reaction method can be directly and easily written on paper using different kinds of handy tools such as ball-pen, brush pen and so on. In this way, people can write out various shapes and patterns conveniently on the flexible substrates according to demands of the target electrical devices owing to the excellent wettability of the liquid metal ink with other materials. The only difference between the present approach and the conventional writing or drawing in daily life is that the liquid metal conductive ink was used here to substitute the water like ink. Figure 6A shows the conductive text written on typing paper by a brush pen dipping a certain amount of GaIn_10_-based liquid metal ink. The word width of the conductive text printed on the typing paper depends on the writing pressure and the tip size of the writing brush or pen. In the present demonstration experiment, they are approximately 1mm wide as shown in Figure 6A. This approach supplies a wonderful material which can be directly written on flexible substrates and make flexible electronics easily and immediately.

A linear array of 9 liquid metal electrodes spaced about 1.5 cm apart and written on the paper using a brush pen dipping GaIn_10_-based liquid metal ink is presented in Figure 6B and Figure 6C, which exhibits excellent adhesion features with paper surface with a bend state. As is known, the metal ink keeps at semi-liquid state around room temperature and therefore will not solidify after writing to a substrate unless it was subjected to an appropriate cooling before use. Hence, the metal sometimes may smear out during mechanical stress. As a remedy, a polyvinyl chloride (PVC) thin-film can be covered on the conductive lines as shown in Figure 6D and Figure 6E, which would protect the conductive lines effectively.

However, the present metal ink in fact also implies very attractive features which conventional electronics cannot offer. For example, since the GaIn_10_-based liquid metal ink remains in liquid state at room temperature and is written on papers as thin conductive lines with the thickness of micron dimensions, it cannot be broken off easily even under frequent bending given appropriate designing. Such ink written on paper shows a predominant mechanical flexibility which is a most important merit in fabricating flexible electronics. Overall, the present method provides a start up principle towards direct writing of electronics. In the near future, more directly printable metal inks with varied melting points can still be developed for different specific application areas.

**Figure 7:**
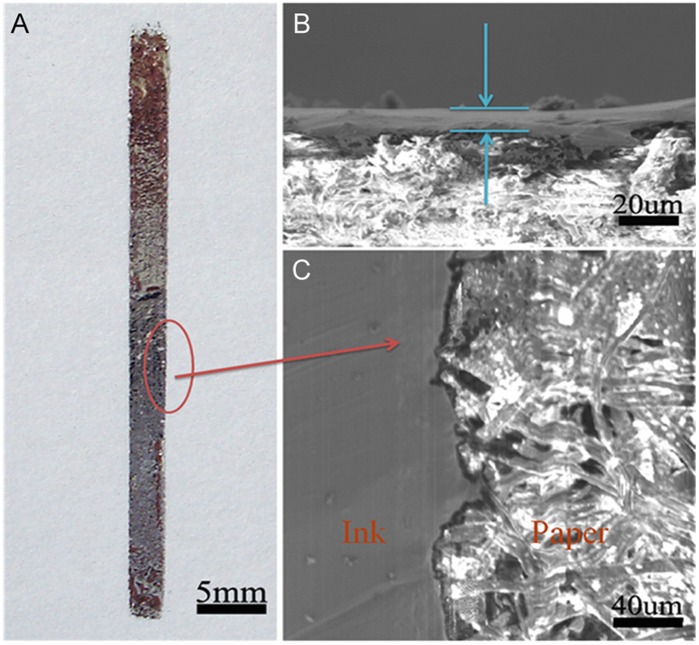
Optical image and SEM graphs of conductive line written on typing paper by using GaIn10-based liquid metal ink. (**A**) Optical image of conductive line written on typing paper by using GaIn_10_-based liquid metal ink. (**B**) SEM graph of thickness of conductive line and paper and (**C**) SEM graph of surface topography of conductive line written on paper.

Figure 7A shows the optical image of conductive line with the width of about 2mm written on typing paper by brush pen. And Figure 7B and Figure 7C show the SEM graphs of cross-section and surface topography of the conductive line, respectively. It can be observed that the liquid metal conductive line is adhered closely to the surface of paper which has a porous structure. The conductive line directly written on paper is more uniform and is about 10 µm thick. The SEM graphs of cross-section and surface topography of the conductive line well proves the excellent wettability of metal ink with the typing paper again.

### Temperature Dependent Electrical Resistivity of Metal Ink

As a kind of special metal ink expecting to be used in future electronics, the electrical property of the present liquid metal ink with 0.026 wt% oxygen as a function of temperature is another important issue which should be clarified. The results measured by the four point probe method have been plotted in Figure 4B. It can be observed that the electrical resistivity of GaIn_10_-based liquid metal ink increases nonlinearly with the temperature, which may partially be attributed to the thermal vibration of atomic nucleus. As is well known, according to the metal conductivity theory, with the increase of the temperature, the atoms in metals will move severely, the vibration amplitude then increases and the structure turns to be more disordered. This would disturb the movement of valence electron, thereby increasing the electrical resistivity of GaIn_10_-based liquid metal [36].

The electrical resistivity of GaIn_10_-based liquid metal ink can be calculated as 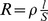, where, *R* is the experimental data of resistance, *ρ* is the electrical resistivity, *l* is length of the sample and *S* is the area of the sample. Hence, the electrical resistivity of the sample can be determined as 34.5 µΩ·cm at 297 K. Clearly, the GaIn_10_-based liquid metal ink with more than 99 wt% GaIn_10_ alloy shows pretty high electrical conductivity. This promises its bright future to serve as electrical conductors and interconnects for many potential applications in electrical devices.

### Drawing of Functional Circuits on Silica Gel Plate

**Figure 8:**
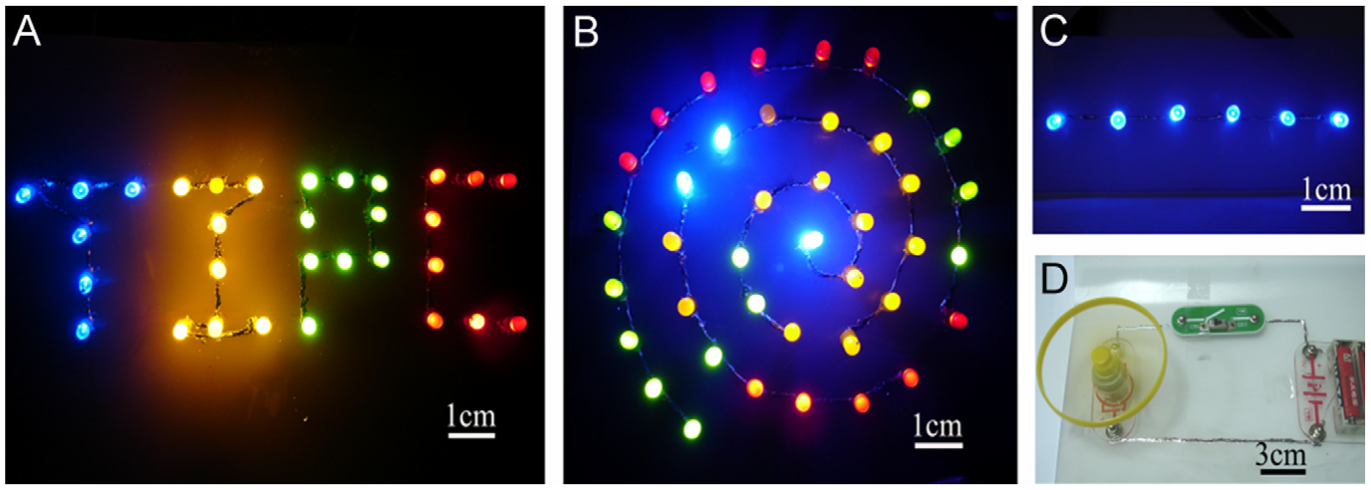
Optical images of LEDs and a fan with GaIn10-based liquid metal ink as electrical interconnects written on silica gel plates. (**A**)-(**C**) Optical images of a flexible substrate display containing LED arrays on a silica gel plate. (**D**) A working fan with GaIn_10_-based liquid metal ink served as electrical interconnects.

To demonstrate the device fabrication capabilities offered by the present approach, we produced multi-color LED displays on silica gel plate as shown in Figure 8. The LEDs are adhered to the flexible substrates by using a drop of superglue placed on the back of each LED. They are then connected to the printed GaIn_10_-based liquid metal electrodes by depositing a drop of GaIn_10_-based liquid metal at each terminal. As examples, an interconnect design with “TIPC” abbreviated from “Technical Institute of Physics and Chemistry” as shown in Figure 8A are produced by illuminating a total of 31 LEDs with four different colors: 6 blue, 8 orange, 9 green and 8 red. Further, the LEDs designed as curvilinear (Figure 8B) and linear (Figure 8C) circuits are composed of different colors which can be integrated into an array illuminated by using a DC power source, although the voltage and current ratings of these LEDs differ by color.

Figure 8D shows a working fan with GaIn_10_-based liquid metal ink serving as electrical interconnects and two 1.5 V batteries served as power source. In the same way, the components of the circuit including a fan, a switch and two batteries are fixed on the substrate with a drop of superglue. Clearly, the liquid metal ink opens the way to directly write functional electrical devices on different substrate materials with a variety of desired lines and patterns. The new method and basic principle has generalized purpose and can be extended to more industrial and business areas, even daily life.

## Discussion

As disclosed by the present fundamental experiments, the content of the gallium oxides in GaIn_10_-based liquid metal ink can efficiently adjust the viscosity of the metal ink which significantly affects the final performance of the circuit lines and patterns written directly on the paper. According to various specific requirements, one can produce the electrical conductors and interconnects with desired sizes spanning from macro to even nano meter scale by controlling the viscosity of metal ink. Beebe *et al.* have used lithographic methods to fabricate microfluidics which are creating new fabrication challenges and finding new applications in biology, chemistry, and materials science [37], [38]. It is believed that the liquid metal ink directly written on different substrates can also be efficiently applied into these fields for fabricating micro and nano electronics devices.

**Figure 9:**
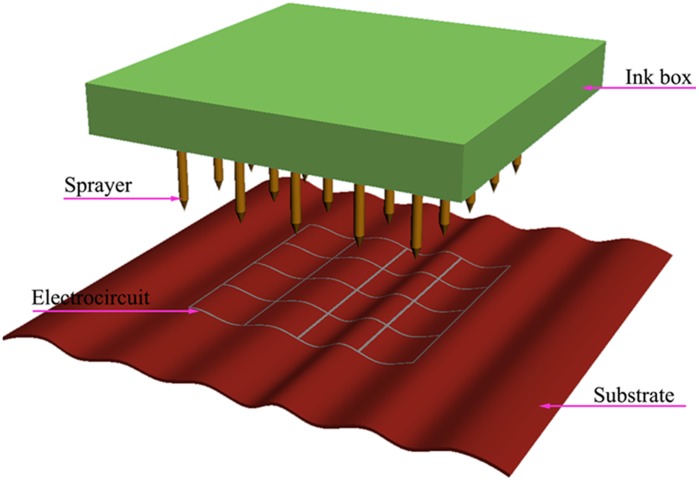
A sketch map of metal ink printer. It can easily and directly print desired electrical lines and patterns on different substrate materials using GaIn_10_-based liquid metal ink.

As is well known, direct-write dip-pen lithography is reported as a potentially useful tool for creating and functionalizing nanoscale devices [39]. Molecules are delivered from the AFM tip to a solid substrate of interest via capillary transport. The only pity is that there lacks of electrical ink at the old days. Following the principle of the current metal ink and the dip-pen lithography, we build a sketch map of printer which can easily and directly print desired electrical lines and patterns on different substrate materials using the GaIn_10_-based liquid metal ink as shown in Figure 9. This suggests a revolutionary way, which is extremely simple however rather powerful, for making expected functionalization of electronic devices prepared by integrating conventional lithographic methods. In addition, GaIn_10_-based liquid metal ink can also play an important role in the well known soft lithography area as opened by Xia and Whiteside [40]. For that contact printing, the liquid metal ink can easily cover the designed patterns with a thin film due to its better wettability with the matched materials and then be easily transported and printed on another desired substrate so as to form hybrid metal-non metal objects.

Further, it is natural to adopt the GaIn_10_-based liquid metal ink for fabricating more complex electrical devices, such as 3D antennas, thin film transistors, electronic solar cell arrays, radio-frequency identification (RFID) tags, flexible batteries and so on. Many functional electrical devices can be made based on the basic concepts enabled by the present paper. If one wishes to modify the physical or chemical properties of the liquid metal ink materials, choosing an appropriate component of alloys or loading selected nano-particles into them so as to make specific compounds will solve the problem. For example, the melting point of gallium-based alloys can be adjusted from several centigrade to several hundred centigrade by changing the components of alloys, which can meet the requirements in different application fields. Unlike GaIn_10_-based liquid metal ink, a heating system should also be installed into the pen to melt the alloys so that they can be written directly on the substrates. Clearly, further optimization of the metal ink into highly conductive or semi-conductive properties and through combination with other printable non-metal materials will bring a new revolution on flexible electronics. An exciting fact in incubation is that people even children without experiences on circuit fabrication can easily write electrical devices on paper or any other desired substrates in the near future.

In summary, the present article demonstrated for the first time the low-cost and direct writing of flexible electronics through the new generation conductive ink, the GaIn_10_-based liquid metal composed of 0.026 wt % oxygen and more than 99 wt % GaIn_10_ alloy. Such material remains in liquid state at room temperature and can be directly written on many soft or rigid materials owing to the well controllable wettability. The thickness of conductive lines can reach about 10 µm. The pretty high electrical conductivity of the ink was measured as 34.5 µΩ·cm at 297 K using four point probe method, which promises its potential future as electrical conductors and interconnects in many electrical devices. As a basic strategy, the present method can also be extended to make more functional and flexible electrical devices, which are expected to be important in a wide range of emerging areas.
